# 

**Published:** 1903-12-12

**Authors:** 


					I * *.* \ ? \ ** V
" . - \ A * i .< ? v * * * 4 1
^UPGEON GENERALS OFF
No. 898. Vol. XXXV. [SKSi?] Saturday, Dec. 12th, 1903. [s " ] PRICE THREEPENCE.

				

